# Use of a TaqMan Array Card for identification of enterotoxins and colonization factors directly from stool samples in an enterotoxigenic *E. coli* vaccine study

**DOI:** 10.1128/spectrum.01870-24

**Published:** 2025-02-11

**Authors:** Jie Liu, T. Sakari Jokiranta, Nils Carlin, Suzanne Stroup, Jixian Zhang, Bjorn Sjostrand, Ann-Mari Svennerholm, Eric R. Houpt, Anu Kantele

**Affiliations:** 1 School of Public Health, Qingdao University 12593 , Qingdao, China; 2 Department of Bacteriology and Immunology, Medicum, University of Helsinki, Helsinki, Finland; 3 Scandinavian Biopharma, Solna, Sweden; 4 Division of Infectious Diseases and International Health, University of Virginia, Charlottesville, Virginia, USA; 5 Gothenburg University Vaccine Research Institute, Department of Microbiology and Immunology, Institute of Biomedicine, University of Gothenburg, Gothenburg, Sweden; 6 Meilahti Vaccine Research Center MeVac, University of Helsinki and Helsinki University Hospital, Helsinki, Finland; Houston Methodist Hospital, Houston, Texas, USA

**Keywords:** enterotoxigenic *E. coli*, ETVAX, TaqMan Array Card, Amplidiag, enterotoxin, colonization factor, travelers’ diarrhea

## Abstract

Enterotoxigenic *Escherichia coli* (ETEC) is a leading cause of childhood and travelers’ diarrhea. The vaccine candidate ETVAX encompasses several ETEC colonization factors (CFs) with a hybrid LT (heat-labile toxin)/cholera toxin B subunit adjuvanted with a double-mutant LT. Stool samples from a Phase 2b ETVAX trial were tested by a PCR-based customized TaqMan Array Card (TAC), including three ETEC toxin genes (LT and heat-stable toxins, STh and STp) and 18 ETEC CFs. Stool samples were also tested with the molecular platform Amplidiag and culture, followed by GM1-enzyme-linked immunosorbent assay (ELISA) and inhibition GM1-ELISA for LT and ST and dot blot for CFs of ETECs identified among six culture isolates (maximum). Compared with Amplidiag, TAC yielded 89.4% sensitivity (320/358) and 96.4% specificity (405/420) for ETEC detection. The two methods demonstrated a good quantitative correlation (quantification cycle *R*^2^ = 0.827, *P* < 0.05). Compared with culture, TAC and Amplidiag each exhibited 96.8% (184/190) sensitivity and identified an additional of 151 and 174 PCR positives in 588 culture-negative stools, respectively. The concordance of stool TAC versus ELISA of ETEC colonies for LT and STh/STp was 85.5% (165/193). TAC demonstrated 98% sensitivity and 92% specificity versus the dot blot results of 793 colonies for the ETVAX CFs CFA/I, CS3, CS5, and CS6. Overall ETEC was detected by TAC in 335 (43.1%) and by Amplidiag in 358 (46.0%) of specimens compared to 190 (24.4%) by culture. We conclude that molecular diagnostic approaches of TAC or Amplidiag increase the detection of ETEC compared with culture, and TAC can also provide vaccine-subtype ETEC data.

**CLINICAL TRIALS:**

This study was registered with ClinicalTrials.gov as NCT03729219.

**IMPORTANCE:**

Enterotoxigenic *Escherichia coli* (ETEC) is an important cause of childhood and travelers’ diarrhea. Vaccines in development utilize specific toxins and colonization factors (CFs) as antigens. Therefore, clinical microbiologic diagnostic methods are needed to discriminate specific toxins and CFs, both for vaccine trials and to guide epidemiology. In this work, we assessed the diagnostic performance of several methods for ETEC: a PCR-based customized TaqMan Array Card (TAC) and the molecular platform Amplidiag on stool and *E. coli* culture, followed by GM1-enzyme-linked immunosorbent assay for toxins and dot blot for CFs. Stool samples from a Phase 2b ETEC vaccine trial were used. Overall, ETEC was detected by TAC in 335 (43.1%) and by Amplidiag in 358 samples (46.0%) compared to 190 (24.4%) by culture. TAC additionally provided CF data with 98% sensitivity and 92% specificity. We conclude that the molecular diagnostic approaches of TAC or Amplidiag increase the detection of ETEC compared with culture.

## INTRODUCTION

Enterotoxigenic *Escherichia coli* (ETEC) is one of the most common causes of diarrhea in children in low- and middle-income countries (1–3) and a leading cause of travelers’ diarrhea (4, 5). It is also associated with childhood mortality (6).

In the intestine, ETEC first adheres to the epithelial cells via proteinaceous surface polymers termed colonization factors (CFs), and then secretes enterotoxins (7). To date, 29 different CFs have been identified, with CFA/I and CS1-CS6 being the most common. ETEC enterotoxins include heat-labile toxin (LT) and heat-stable toxin (ST). There are two types of ST: STa found in both humans and livestock and STb exclusive in livestock. STa encompasses two subtypes, STh and STp. Human ETEC is characterized by the presence of either LT and/or ST (STh or STp) with or without one or more CFs.

ETEC vaccines are under development, including the oral vaccine ETVAX (Scandinavian Biopharma, Solna, Sweden). This vaccine utilizes recombinant *E. coli* strains that over-express ETEC colonization factors CFA/I, CS3, CS5, and CS6 combined with a hybrid LT/cholera toxin B subunit (LCTBA) adjuvanted with a double-mutant LT (8). A recent Phase 2b double-blinded, randomized, placebo-controlled trial demonstrated that ETVAX was safe and strongly immunogenic (9).

When evaluating the efficacy of ETEC vaccines, it is desirable to accurately assess not only ETEC but also the specific toxins and CFs, as this enables distinct efficacy calculations against vaccine-preventable strains and strains not covered by vaccine targets. However, the accurate identification of enterotoxins and colonization factors is cumbersome. Conventionally, stool culture is performed to obtain a number of *E. coli* isolates, followed by enterotoxin detection by enzyme-linked immunosorbent assay (ELISA) or PCR. Colonization factors have historically been identified with phenotypic methods using polyclonal or monoclonal antibodies (MAbs) against various CF epitopes (10). A higher throughput method could use PCR on direct stool specimens. We and others have developed PCR assays for ETEC toxins and colonization factor detection that perform well on both cultured isolates and direct stool (11–13). Additionally, we have developed a quantitative PCR (qPCR)-based TaqMan Array Card (TAC) for the molecular diagnosis of diarrhea (2–5).

Here, we applied a customized TAC, which included three ETEC toxin genes, 18 ETEC CFs, and other pathogens, to a large ETVAX field trial (9). The diagnostic performance of this customized TAC was examined and compared with the combination of stool culture, molecular detection, GM1-ELISA and inhibition GM1-ELISA, and dot blot.

## MATERIALS AND METHODS

### Study design

The study explored real-time PCR-based TAC for assessing ETEC and its toxins and CFs conducted within the OEV123 clinical trial on ETVAX (see below). The performance of TAC in detecting ETEC was compared with a commercial multiplex PCR-based bacterial GE assay of Amplidiag (Mobidiag, Espoo, Finland; later acquired by Hologic, Inc.) directly from stools. TAC results on ETEC toxins and CFs were compared to results obtained from PCR-confirmed culture isolates for toxin production by GM1-ELISA and inhibition GM1-ELISA and the presence of CFs by dot blot.

The OEV123 study protocol was approved by the Ethics Committee of HUS Helsinki University Hospital (HUS/2231/2016) and the Finnish Medicines Agency (EudraCT 2016-002690-35). Written informed consent was obtained from all participants.

### OEV123 clinical trial

OEV123 was a Phase 2b randomized, double-blinded, placebo-controlled trial among Finnish travelers to Benin, West Africa. The study design, safety, and immunogenicity data have been published earlier (9). OEV123 had 749 volunteers aged 18–65 years, who were randomized 1:1 to receive either two doses of ETVAX or placebo. All volunteers visited Benin for 12 days (9).

### Specimens

All OEV123 participants provided both routine stool samples (pre-travel, Day 4 onsite, and post-travel) and samples (3rd and 4th stool) during each diarrheal episode occurring either in Benin or during a follow-up period of 6 days post-travel. In total, 116 routine and 662 diarrheal stool samples were collected from 616 adult travelers to Benin between 31 May 2017 and 15 April 2019 (9). The majority of the diarrheal samples (81.6%, 540/662) were collected in Benin, with 122 obtained post-travel.

Each stool sample was collected into two primary stool eNAT tubes (Copan, Italy) and a fecal swab tube (Copan, Italy). The fecal swab was used to culture the sample onto culture media plates within 12 h of sample collection at Villa Karo Research Centre in Benin or at United Medix Laboratories, Ltd. (UML) in Helsinki, Finland for the samples collected after leaving from Benin. The eNAT samples (containing either primary feces or the pooled colony sample) and the cultured isolates in cryobead tubes were stored at −80°C and transported on dry ice with a temperature monitor to United Medix Laboratories, Ltd. (UML) in Finland. The other primary stool eNAT aliquots were transported on dry ice with a temperature monitor to the University of Virginia in the United States. Samples were stored at −80°C until testing.

### Stool culture followed by Amplidiag, ELISA, and dot blot

The culture medium plate used to isolate the *E. coli* colonies was ChromID CPS Elite (BioMérieux). Twelve colonies with the appearance of *E. coli* were isolated from this plate and cultured on separate culture medium plates (CPS Elite), followed by storing in cryobead tubes (each with 25 cryobeads; Biomérieux). Six of these colonies were used in this study, and a pooled eNAT sample of these six colonies was prepared for each fecal sample. To detect ETEC in the pooled eNAT samples, the Amplidiag Easy Extraction Kit was used to isolate nucleic acids from the eNAT samples, followed by the Amplidiag bacterial GE test on a Bio-Rad CFX96 real-time PCR system per the manufacturer’s instructions (14). If a pooled eNAT tube was positive for ETEC, two cryobeads from each of the six cryobead tubes (each prepared from a single bacterial colony isolate) were placed to an eNAT tube. These eNATs for the six colony isolates were tested individually using the Amplidiag bacterial GE test (Mobidiag, Espoo, Finland) (14). For each ETEC positive bacterial isolate eNAT sample, a corresponding cryobead was cultured on CPS Elite plate, and the species identity was confirmed with a VITEK Mass Spectrometry–Matrix-assisted Laser Desorption Ionization Time-of-Flight System (BioMérieux). Each colony was stored in tryptic soy deep agar (Tammer BioLab, Ltd., Finland) and sent to University of Gothenburg, Sweden for GM1-ELISA and inhibition GM1-ELISA LT and ST tests, STh and STp by PCR, and MAb-based dot blot tests for CFs (15).

### Direct stool testing by Amplidiag

To detect ETEC, an Amplidiag Easy Extraction Kit in Amplidiag platform was used to isolate nucleic acids from the eNAT samples, followed by the Amplidiag bacterial GE test on a Bio-Rad CFX96 Real-time PCR System per the manufacturer’s instructions (Mobidiag, Espoo, Finland).

### Direct stool testing using the TaqMan Array Card

Total nucleic acid was extracted from 200 µL of stool samples stored in eNAT using the QIAamp Fast DNA Stool Mini Kit (Qiagen, Hilden, Germany). Briefly, the stool samples were pretreated with bead beating and incubation at 95°C to improve the nucleic acid yield, followed by the standard protocol recommended by the manufacturer. The nucleic acid was eluted in 200 µL and stored at −80°C. External controls, phocine herpesvirus (PhHV) and MS2 bacteriophage, were spiked into each sample during the initial lysis step to monitor the extraction and amplification efficiency. One extraction blank was included per batch of extraction to monitor a possible contamination. A customized TaqMan Array Card was formulated to detect ETEC toxins (LT, STh, and STp), ETEC colonization factors (CFA/I, CS1, CS2, CS3, CS4, CS5, CS6, CS7, CS8, CS12, CS13, CS14, CS17/19, CS18, CS20, CS21, and CS22), as well as other 19 enteric pathogens with potential association with travelers’ diarrhea (Fig. S1; Table S1). ETEC is defined as the detection of either LT, STh, or STp and assigned with the lowest Cq (quantification cycle) of the three toxin genes. The 100 µL PCR reaction consisted of 50 µL of AgPath one-step RT-PCR reagent (Life Technologies, Carlsbad, California), 4 µL of enzyme mix, and 40 µL of total nucleic acid extract. The real-time PCR was performed with the cycling condition of 20 min at 45°C, 10 min at 95°C, 40 cycles of 15 s at 95°C, and 1 min at 60°C. The positivity was determined with a Cq cut-off of 35 for all the targets. Additionally, associated enterotoxins were required to be within 3 Cqs of each other, as were associated CF(s) within 3 Cqs of the enterotoxin.

### Analysis of ETEC toxin production and CFs by ELISA and dot blot

GM1-ELISA and inhibition GM1-ELISA to detect LT and ST, respectively, were performed as previously described (15). In short, *E.coli* colonies were incubated in GM1-coated microtiter plates containing Luria Bertani broth (LB) supplemented with lincomycin and glucose at 37°C overnight. Released LT bound to the solid-phase GM1 was detected by incubation with an anti-LT MAb, followed by incubation with an anti-mouse IgG horseradish peroxidase conjugate and development with enzyme substrate. For detection of ST based on an inhibition ELISA, the LB colony cultures (retrieved from the GM1-coated plate used for LT detection) mixed with an anti-ST MAb were incubated in GM1-coated plates with bound ST-CTB conjugate. Inhibition of the binding of the anti-ST MAb to the solid-phase ST-CTB was assessed as for the LT MAb. ST positive colonies were further analyzed by PCR to distinguish between STh and STp (15). Dot blot tests to detect CFs expressed on the *E. coli* colonies were performed by applying dots of the toxin-positive colonies cultured on CF agar overnight on nitrocellulose sheets; strips with the dots were subsequently developed by incubation with 12 different MAbs against CFA/I, CS1-CS6, CS7, CS8, CS12, CS14, and CS17 (one MAb per strip), followed by anti-mouse IgG enzyme conjugate end enzyme substrate (15). Positive colonies were visualized as black dots on the strips.

### Statistics

Correlation was tested by regression analysis using the analysis of variance test. qPCR Cq values were compared with the Mann–Whitney *U* test between culture-positive and -negative samples. Receiver operating characteristic (ROC) analysis was used to determine the TAC Cq cut-offs of enterotoxin genes versus culture. Two-tailed *P* values were calculated, with values < 0.05 considered statistically significant. All analyses were performed using IBM SPSS version 27.

## RESULTS

### Detection of ETEC enterotoxin genes

**Fig 1:**
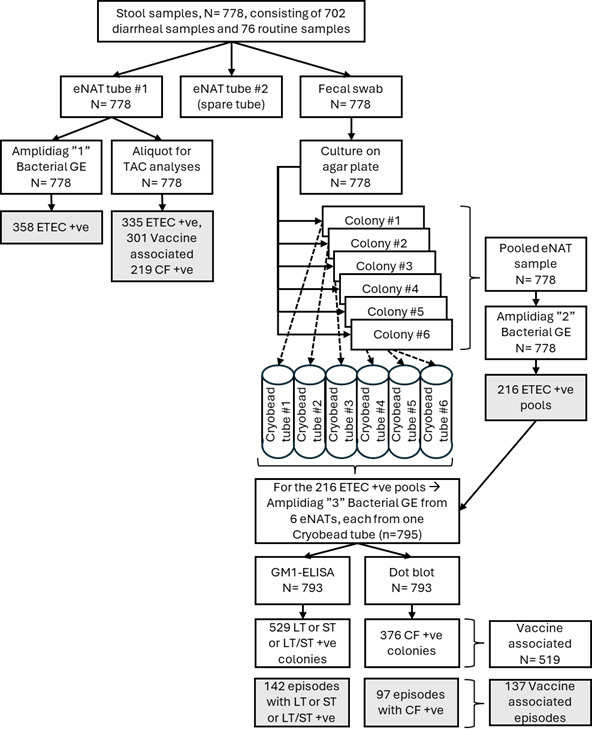
Testing algorithm. TAC and Amplidiag were performed directly on stool samples taken to eNAT. Stool culture was performed, and six colonies with the appearance of *E. coli* were isolated and stored in cryobead tubes. A pool of the six isolates was formed in eNAT. The pooled samples were subjected to PCR detection of ETEC with Amplidiag. In case the pool was positive, individual colonies were tested with Amplidiag. Although 358 fecal specimens were positive with Amplidiag directly from stool samples, only 216 of the colony pools were positive with Amplidiag, and, out of these 216 samples, a total of 795 ETEC colonies could be isolated (average of 3.7 colonies/sample).The ETEC-positive colonies were finally subjected to enterotoxin detection with GM1 ELISA and CF identification with dot blot. Vaccine-associated strains were those with ETVAX targets consisting of LT, CFA/I, CS3, CS5, and CS6.

**Fig 2:**
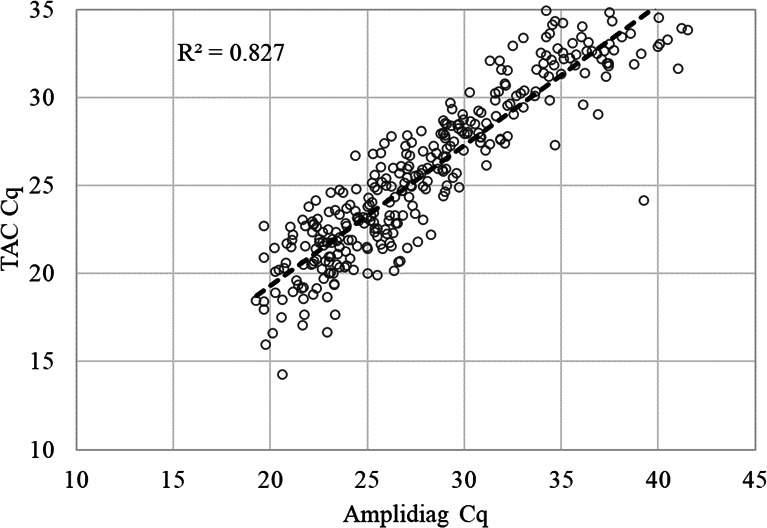
Correlation of ETEC Cq values between TaqMan Array Card and Amplidiag directly on stool samples. if multiple toxins were positive, the lowest Cq of LT, STh, and STp was used.

The qPCR assays utilized on the customized TAC, including those targeting ETEC enterotoxin genes and CFs, have been published previously (2, 11, 16–18). The nucleic acid extraction methodology of stool samples stored in eNAT was evaluated with spiked samples and showed that eNAT had little effect on the yield and quality compared with the usual QIAamp Fast DNA Stool Mini Kit (data not shown). As shown in Fig. 1, among 778 stool samples, 335 (43.1%) were positive for ETEC with TAC, and 358 (46.0%) were positive with Amplidiag; TAC demonstrated 89.4% sensitivity (320/358) and 96.4% specificity (405/420) compared to Amplidiag. The two methods demonstrated a good correlation for Cq values (Fig. 2, *R*^2^ = 0.827, *P* < 0.05). The Cqs of the samples that were ETEC-positive by Amplidiag only or TAC only were significantly higher than those of the samples that were positive by both methods (Amplidiag+/TAC− Cq 37.4 ± 5.0, TAC+/Amplidiag− Cq 32.3 ± 3.8 versus Amplidiag Cq 28.2 ± 5.4 and TAC Cq 25.7 ± 4.6 of TAC+/Amplidiag+, *P* < 0.05). Among the ETEC positive non-diarrheal samples collected, Cq values were significantly higher than those of diarrheal samples [for TAC 28.1 ± 4.0 for non-diarrheal (*n* = 30) versus 25.7 ± 4.8 for diarrheal (*n* = 305), *P* = 0.011; for Amplidiag 32.9 ± 5.3 for non-diarrheal (*n* = 26) and 28.8 ± 6.0 for diarrheal (*n* = 332), *P* = 0.001].

Pooled *E. coli* culture isolates were tested with Amplidiag, and 216 pools from 190 out of 778 samples were ETEC-positive. Compared with these stool culture results, both TAC and Amplidiag from the primary stool eNAT sample demonstrated 96.8% sensitivity (184/190) with an additional 151 (TAC) and 174 (Amplidiag; 95.3% sensitivity and 100 additional detections with Amplidiag positivity defined as Cq < 35) ETEC out of 588 culture negative stools. The stool Cqs of both TAC and Amplidiag demonstrated a quantitative trend as the number of ETEC-positive colonies increased from 0 to 6 (Fig. S2). The Cqs of TAC+/Culture− samples were significantly higher than those of TAC+/Culture+ (29.7 ± 3.9 versus 23.4 ± 3.4, *P* < 0.001). Similarly, the Cq values of Amplidiag+/Culture− samples were significantly higher than those of Amplidiag+/Culture+ (33.1 ± 5.6 versus 25.4 ± 3.6, *P* < 0.001).

**Fig 3:**
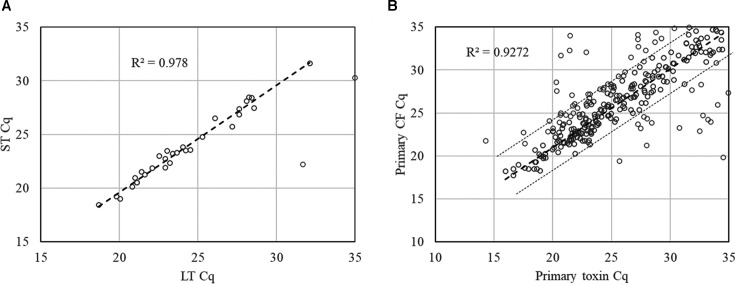
Cq correlation (**A**) between enterotoxin LT and ST (either STh or STp) and (**B**) between enterotoxins and primary colonization factors. The thick dashed line indicates the correlation curve, while the thin dashed lines indicate the 3 Cq window.

Among 190 samples with ETEC-positive colony pools, either LT or ST was detected with GM1-ELISA from individual colonies of 178 samples. TAC showed 98.9% (176/178) concordance with the ELISA results on ETEC colonies. TAC assays further distinguished LT from ST. The lower Cq of the three enterotoxins was used as the ETEC Cq in the analyses. Since an excellent correlation was observed between LT Cq and ST Cq (either STh or STp) for culture isolates possessing both LT and ST (Fig. 3A, R^2^ = 0.978), LT/ST was assigned when the delta Cq between LT and ST was within 3, otherwise LT-only or ST-only was called. With this assignment, TAC identified primary enterotoxin types in the stool samples, yielding 80.0% (56/70), 93.5% (58/62), and 83.6% (51/61) concordance for culture-positive LT/ST, LT-only, and ST-only ETEC, respectively. Most discrepancies were missing TAC detections of LT (*n* = 10) for LT/ST and additional TAC detections of LT (*n* = 9) for ST-only. There was no Cq difference in the discrepant results.

### Quantitative identification of primary colonization factors

Previous work on both ETEC isolates and stool samples (11) has demonstrated a tight correlation between enterotoxin Cqs and CF Cqs. Therefore, a CF was considered associated with ETEC when its Cq value was within 3 units of the enterotoxin Cq value (Fig. 3B, *R*^2^ = 0.927, *P* < 0.05). CF(s) were determined for each ETEC sample with this criterion applied. Table 1 presents the comparison between TAC and dot blot results. TAC demonstrated overall 88.4% sensitivity and 98.0% specificity versus dot blot. For the CF candidates used in the ETVAX vaccine, namely, CFA/I, CS3, CS5, and CS6, the sensitivity of TAC was 98%, and the specificity was 92% (*n* = 1025). TAC also detected CS13 (2.0%), CS15 (1.7%), CS18 (0.3%), CS20 (5.5%), CS21 (10.8%), and CS22 (9.0%), which were not evaluated by dot blot. CS4 and CS7 were not detected. Altogether, one or more CFs was detected in 63.8% (219/343) of the ETEC positive samples by TAC.

**TABLE 1:** Comparison of the ETEC colonization factor detection between TAC directly on stool and dot blot on cultured isolates^*a*^^,^^*b*^

	TAC+/dot+	TAC+/dot−	TAC-/dot+	TAC−/dot−	Sensitivity, %	Specificity, %
CFA/I	12	1	0	192	100	100
CS1	6	3	1	195	86	99
CS2	20	6	0	179	100	97
CS3	28	6	1	170	97	97
CS5	1	0	1	203	50	100
CS6	61	19	11	114	85	86
CS8	0	0	0	205	–	100
CS12	7	0	4	194	64	100
CS14	2	2	0	201	100	99
CS17/19	0	0	0	205	–	100

^*a*^ This analysis was limited to the culture-positive samples that underwent CF testing.

^*b*^ TAC+, CF positive by TAC on direct stool; TAC−, CF negative by TAC on direct stool; Dot+, CF positive by dot blot on ETEC isolates; Dot−, CF negative by dot blot on ETEC isolates.

### Determination of vaccine-associated ETEC

Table 2 summarizes the overall concordance of the detection of ETVAX targets between TAC and culture-based isolation of *E. coli* strains, followed by Amplidiag for detection of ETEC and GM1-ELISA for differentiation of enterotoxins and dot blot for CFs. Using culture as the reference, TAC identified 59/61 (97%) of LT ETEC, 56/70 (80%) of LT/ST ETEC, 51/60 (85%) of ST ETEC, and 139/191 (73%) of toxin and CF combinations. Examining the entire sample set of 778 participants, 93.3% (166/178) of the ETEC culture positives was from vaccine-preventable ETEC cases (LT with or without CFA/I, CS3, CS5, or CS6), while TAC detected 301/335 cases (89.9%). The extra TAC detections of vaccine-preventable ETEC were generally of similar toxin and CF combinations as the culture-based detections, predominantly LT, LT/ST, LT/CS6, LT/ST/CS3, or ST/CS6.

**TABLE 2:** Detection of ETEC enterotoxins and colonization factors targeted by ETVAX^*a*^^,^^*c*^

	Culture–E–D^*b*^	Toxin	LT	LT	LT, ST	LT, ST	LT, ST	LT, ST	LT, ST	ST	ST	ST	ST	ST	ETEC negative	ELISA/ dot blot negative
TAC		CF	–	CS6	–	CFA/I	CS3	CS3, CS6	CS6	–	CFA/I	CS3	CS5, CS6	CS6		
Toxin	CF															
LT	–		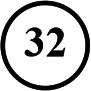	0	**4**	0	0	0	0	0	0	0	0	0	0	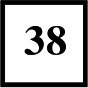
LT	CS3		**1**	0	0	0	0	0	0	0	0	0	0	0	0	0
LT	CS6		**7**		0	0	0	0	0	**1**	0	0	0	0	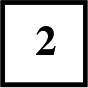	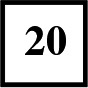
LT, ST	–		0	0		0	**1**	0	**1**	**2**	0	0	0	**1**	0	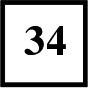
LT, ST	CFA/I		0	0	0		0	0	0	0	0	0	0	0	0	0
LT, ST	CS3		0	0	**1**	0		**2**	0	0	0	**1**	0	0	0	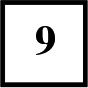
LT, ST	CS3, CS6		0	0	0	0	**1**	0	0	0	0	0	0	**2**	0	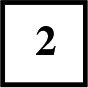
LT, ST	CS6		0	**2**	**1**	0	0	0		0	0	0	0	**2**	0	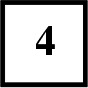
ST	–		0	0	**2**	0	0	0	**1**		0	0	0	**6**	**1**	**21**
ST	CFA/I		0	0	0	**3**	0	0	0	0		0	0	0	0	0
ST	CS5, CS6		0	0	0	0	0	0	0	0	0	0		0	0	0
ST	CS6		0	0	0	0	0	**1**	**3**	**4**	0	0	0		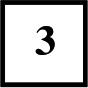	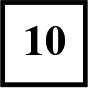
ST	CFA/I, CS6		0	0	0	0	0	0	0	0	0	0	0	**1**	0	0

^*a*^ The columns show the detection results by culture combined with GM1-ELISA for enterotoxin detection and differentiation and dot blot for CF detection. The rows show the detection results by TAC. The circled numbers indicate the concordant results between TAC and culture (six predominant isolates) combined with GM1 ELISA for enterotoxin detection and dot blot for CF detection of Amplidiag screening-positive samples. The numbers in the box indicate the additional detection for ETVAX targets by TAC.

^*b*^ Culture (six predominant isolates) combined with GM1-ELISA for enterotoxin detection and differentiation and dot blot for CF detection.

^*c*^ – No CF detected.

## DISCUSSION

This work evaluated the performance of a one-step qPCR approach using a TaqMan Array Card compared with a multi-step culture-based regimen to detect ETEC subtypes in stool. The molecular approaches on direct stool, whether TAC or the commercial Amplidiag molecular method, exhibited high sensitivity for detecting ETEC compared with culture. Specifically, TAC and Amplidiag exhibited a sensitivity of 96% compared to culture methods. The molecular methods also yielded additional detections of ETEC, thereby detecting ETEC in 335 (43.1%; TAC) or 358 (46.0%, Amplidiag) of samples overall. This high sensitivity of molecular methods versus culture on stool specimens is widely noted (18). The ETEC detections by TAC and Amplidiag were highly correlated. Increasing the number of *E. coli* colonies tested for ETEC can enhance the sensitivity of culture (19) but substantially increases the workload.

Among the 190 culture-positive ETEC, GM1-ELISA and inhibition GM1-ELISA detected 70 (36.8%) as positive for both ST and LT, of which TAC identified 56, plus excessive 12 LT/ST detection. These molecular versus phenotypic discrepancies could reflect the absence of enterotoxin gene expression or phenotypic silencing, which can occur during storage and recultivation (15), as noted by a previous report where toxin types were phenotypically detected in only 63% of PCR-positive ETEC strains (20). As the Amplidiag bacterial GE panel does not differentiate LT from STh or STp, this analysis could not be performed with Amplidiag. Worth noting is that LT and ST toxins could be present in distinct colonies from a given sample. Such mixed infections can only be detected with a culture-based approach and is not possible with whole-stool PCR.

Of the 335 ETEC positive samples detected by TAC, 65.4% (219/335) was positive for one or more CFs. Both TAC and dot blot techniques showed that CS6 was the predominant colonization factor across ETEC, with various combinations of enterotoxins. LT-only ETEC possessed CS6 or CS13. CFA/I was highly associated with STh-only ETEC as in other studies (21, 22), and CS14 was also detected in STh-only ETEC. CS3 was mainly detected in LT-STh ETEC often combined with CS1 or CS2 with or without CS21.

Such enterotoxin-CF patterns are important for ETEC epidemiology, vaccine development targets, and the evaluation of cross-protection of vaccines (23). For example, according to the GEMS study, approximately 66% of pediatric diarrhea cases caused by ETEC could be prevented if effective ETEC vaccine candidates based on CFA/I and CS1-CS6 were developed, and the rate would increase to 77% if CS14 was added to CF-based ETEC vaccine candidates (22).

We should note that we did not apply a quantitative Cq cut-off approach to detect ETEC diarrhea in this analysis. We have used such an approach in prior studies of childhood diarrhea in low resource settings to alleviate and address low-level carriage; however, previous traveler’s diarrhea studies have demonstrated a stronger association between ETEC and diarrhea across quantities (4, 5). That said, mixed infections with ETEC and other enteropathogens is possible (2, 3), and a more accurate weighting of the attribution of each pathogen at the patient level could be valuable in a vaccine trial. For instance, in a rotavirus vaccine trial, after taking coinfections of other diarrheagenic pathogens into account, the vaccine efficacy estimate increased by 11.3% (24). Such an approach could be employed for the ETEC vaccine trial design. Similarly, the very high sensitivity of the molecular methods in detecting clinically insignificant amounts of pathogens may also pose a challenge, as the primary focus is on preventing symptoms rather than infection. Consequently, in the OEV123 protocol, the evaluation of efficacy focused on cultured isolates. This work was designed and pre-specified as a diagnostic sub-study and did not include any efficacy analyses. Although the OEV123 protocol did not include TAC for efficacy analyses, possible further exploratory analyses on the correlation between TAC results and efficacy of the vaccine may be of interest.

In summary, a customized TAC on direct stool, as well as the Amplidiag bacterial GE assay, performed well as a one-step test for ETEC. In addition, TAC confirmed the specific toxin profiles and CFs found on culture quite well.

## Data Availability

TaqMan Array Card data is available at https://figshare.com/articles/dataset/ETEC_detection_by_TaqMan_Array_Card_in_ETVAX/28248518?file=51827288.

## SUPPLEMENTAL MATERIAL

Supplemental material spectrum.01870-24-s0001.docx Figures S1 and S2; Table S1.

ASM does not own the copyrights to Supplemental Material that may be linked to, or accessed through, an article. The authors have granted ASM a non-exclusive, world-wide license to publish the Supplemental Material files. Please contact the corresponding author directly for reuse.
