# Supplementary material for: Use of a TaqMan Array Card for identification of enterotoxins and colonization factors directly from stool samples in an enterotoxigenic E. coli vaccine study
Source: Microbiol Spectr. 2025 Feb 11;13(3):e01870-24. doi: 10.1128/spectrum.01870-24 (PMC11878035; doi:10.1128/spectrum.01870-24)
Supplement: Supplemental material — Figures S1 and S2; Table S1. [file spectrum.01870-24-s0001.docx]

**Supplemental Figure S1** The customized TaqMan Array Card utilized in the current study. Each card runs 8 samples. L and R indicated the two separated columns for each sample, containing 24 PCR reaction wells each. A single target (FAM fluorophore) or dual targets (indicated by “&”, labeled with FAM and VIC, respectively) are included in each of reaction.


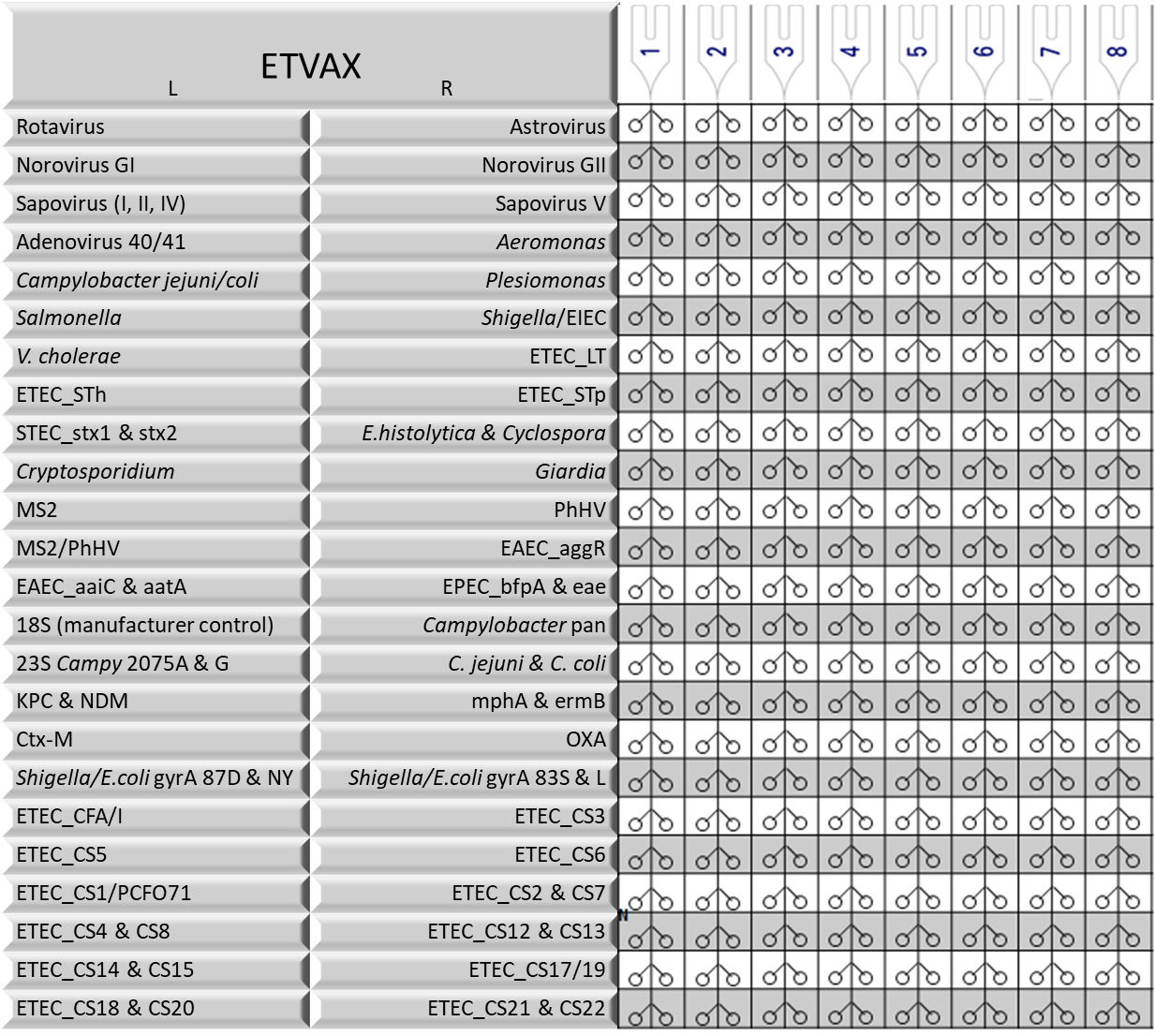


**Supplemental Figure S2** The ETEC Cq distribution of TAC and Amplidiag varied only slightly by the number of culture colonies positive for ETEC. The median and 1^st^/3^rd^ quantile were shown, and the outliers were defined as outside the range between 1st quartile – 1.5*interquartile range and 3rd quartile + 1.5*interquartile range.


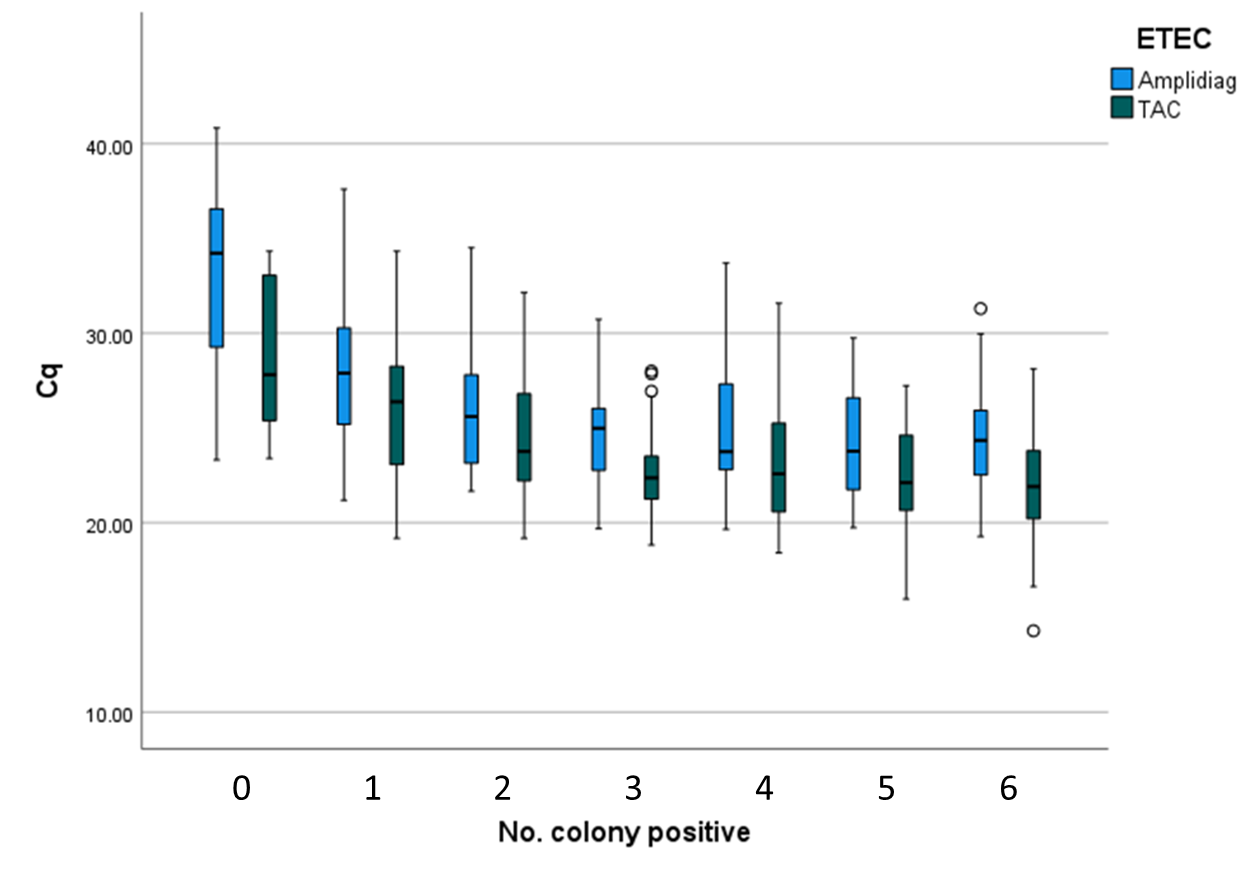


Supplemental Table S1 The primer and probe sequences for the ETEC qPCR assays used in the TaqMan Array Card.

| Target | | Sequences (5’-3’) |
| --- | --- | --- |
| LT | Forward  Reverse  Probe | TTCCCACCGGATCACCAA  CAACCTTGTGGTGCATGATGA  CTTGGAGAGAAGAACCCT |
| STh | Forward  Reverse  Probe | GCTAAACCAGYAGRGTCTTCAAAA  CCCGGTACARGCAGGATTACAACA  TGGTCCTGAAAGCATGAA |
| STp | Forward  Reverse  Probe | TGAATCACTTGACTCTTCAAAA  GGCAGGATTACAACAAAGTT  TGAACAACACATTTTACTGCT |
| CFA/I | Forward  Reverse  Probe | AGCTTATTCTCCCGCATCAAA  GAACATCTGTAAGCTGTGGTGT  ACTCAAGTACATACAAACGATGCA |
| CS1/PCFO71 | Forward  Reverse  Probe | ACTTTGCTTCGAGTGGTGTT  CCCTGATATTGACCAGCTGTTA  CAGAAACTTTCAATCCATGCAGAT |
| CS2 | Forward  Reverse  Probe | TCTGCTCGTATCAATACCCAAGTT  GTGCCAGCGAATGAAACCTCTA  TCTGATCCAAGCAAGACTATTCC |
| CS3 | Forward  Reverse  Probe | GGTCTTTCACTGTCAGCTATGA  CCAAGTTGCATCCAGAGCTG  TGGCATTAAATGTGCTTTCTCCT |
| CS4 | Forward  Reverse  Probe | CTATTCACCTGCGGCAAGTC  GGGGAGTTGTTTTGTAGAATCCA  TCGCAACTAAAGTTCATACAAATGT |
| CS5 | Forward  Reverse  Probe | GCGTGACACGTCAGCTAATATAAAC  AAAGTGATTGCGACTTCCCC  ACCGCAGTAGAAGCAGCTAA |
| CS6 | Forward  Reverse  Probe | GGAGTGGTAAATGCAGGAAACT  GAACAGCGGAATCAATATCTGGA  CTCTGGATGTAAATGTAAATATTGAG |
| CS7 | Forward  Reverse  Probe | TGCTCCCGTTACTAAAAATACGT  CGAACGGGCTGTGATACCTT  CCAATCCGTTCACAAAAGCC |
| CS8 | Forward  Reverse  Probe | ACTGGGAGTATGTGGCAGTTG  TATTGTAGTATTATCAGTAGCAGCCA  CATGTTTACTGCGCCTGCA |
| CS12 | Forward  Reverse  Probe | TTACGTCTCTGATCATGGCTGTTA  TTGTTATTCGCTTGGCCGTT  ATGAATAGCTCAGCCTTCGC |
| CS13 | Forward  Reverse  Probe | GGGACTGCCACAATGAATTT  TGCATACAGAGACGCTCGAT  CACCATAAGCAGCTGCTGAA |
| CS14 | Forward  Reverse  Probe | TCATGGGCAGGGAAGACATT  TACTATTCGAAACACCTGCCG  AGTTGGCGATCTGGGTTTTG |
| CS15 | Forward  Reverse  Probe | CGAAATTGGACAAGCGATG  AGGCGCGAATGTCAAGATTA  CCCATAGTAGGCCACAGTGG |
| CS17/19 | Forward  Reverse  Probe | AGGSAGTTGTAGTGAAGCTGT  GTCACTTTCATCGGAATTTGCGA  CAGTTCTGTCCAATATTATGAAGCCA |
| CS18 | Forward  Reverse  Probe | GGTACCTTAAATGGCCAGCC  TAACAGTACCAGCTTTAACCTGAC  CGCATTGCCCAAAACACCT |
| CS20 | Forward  Reverse  Probe | AGGTATCCAAATCCGCACTG  CATCAGCCAGCACATAGGAA  TCGTCAGTCAGGCTGTCAC |
| CS21 | Forward  Reverse  Probe | GGACCCATTAAGCCTTACTGC  GTTATTACGCACTTCGTCTGGT  TGCAGCACAGTTAGTTCAGC |
| CS22 | Forward  Reverse  Probe | GTCCAACACGCTCATAGCAG  TCCAGCAGGGATATTATCATTTTT  TGCCAGACGCTGGGAATA |
